# A clinical case report of Balamuthia granulomatous amoebic encephalitis in a non-immunocompromised patient and literature review

**DOI:** 10.1186/s12879-023-08228-6

**Published:** 2023-04-18

**Authors:** Jun Liu, Wenjun Zhang, Shanlian Wu, Tianxiang Zeng, Fei Luo, Qiuhua Jiang, Ruijin Yang

**Affiliations:** 1 Department of Neurosurgery, Ganzhou People’s Hospital, Ganzhou, 341000 Jiangxi Province People’s Republic of China; 2 Department of Rehabilitation Medicine, Ganzhou People’s Hospital, Ganzhou, 341000 Jiangxi Province People’s Republic of China; 3 Department of Pathology, Ganzhou People’s Hospital, Ganzhou, 341000 Jiangxi Province People’s Republic of China

**Keywords:** *Balamuthia mandrillaris*, Granulomatous amoebic encephalitis, Non-immunocompromised, Surgical treatment, Effective antibiotics

## Abstract

**Background:**

*Balamuthia* granulomatous amoebic encephalitis (GAE) is a peculiar parasitic infectious disease of the central nervous system, about 39% of the infected Balamuthia GAE patients were found to be immunocompromised and is extremely rare clinically. The presence of trophozoites in diseased tissue is an important basis for pathological diagnosis of GAE. *Balamuthia* GAE is a rare and highly fatal infection for which there is no effective treatment plan in clinical practice.

**Case presentation:**

This paper reports clinical data from a patient with *Balamuthia* GAE to improve physician understanding of the disease and diagnostic accuracy of imaging and reduce misdiagnosis. A 61-year-old male poultry farmer presented with moderate swelling pain in the right frontoparietal region without obvious inducement three weeks ago. Head computed tomography(CT) and magnetic resonance imaging(MRI) revealed a space-occupying lesion in the right frontal lobe. Intially clinical imaging diagnosed it as a high-grade astrocytoma. The pathological diagnosis of the lesion was inflammatory granulomatous lesions with extensive necrosis, suggesting amoeba infection. The pathogen detected by metagenomic next-generation sequencing (mNGS) is *Balamuthia mandrillaris*, the final pathological diagnosis was *Balamuthia* GAE.

**Conclusion:**

When a head MRI shows irregular or annular enhancement, clinicians should not blindly diagnose common diseases such as brain tumors. Although *Balamuthia* GAE accounts for only a small proportion of intracranial infections, it should be considered in the differential diagnosis.

## Background

*Balamuthia* GAE is a peculiar parasitic infectious disease of the central nervous system, and is extremely rare clinically, with a mortality rate of nearly 90% [1]. Clinicians and radiologists have little understanding of the disease, and clinical presentation and neuroimaging examination are non-specific, making it easy to be misdiagnosed and missed. Its diagnosis relies on the presence of amoebic trophozoites on histopathological and etiological examinations. This paper reports clinical data from a patient with *Balamuthia* GAE to improve physician understanding of the disease and diagnostic accuracy of imaging and reduce misdiagnosis. The publication of this case report obtained written informed consent from the patient.

## Case presentation

**Fig. 1:**
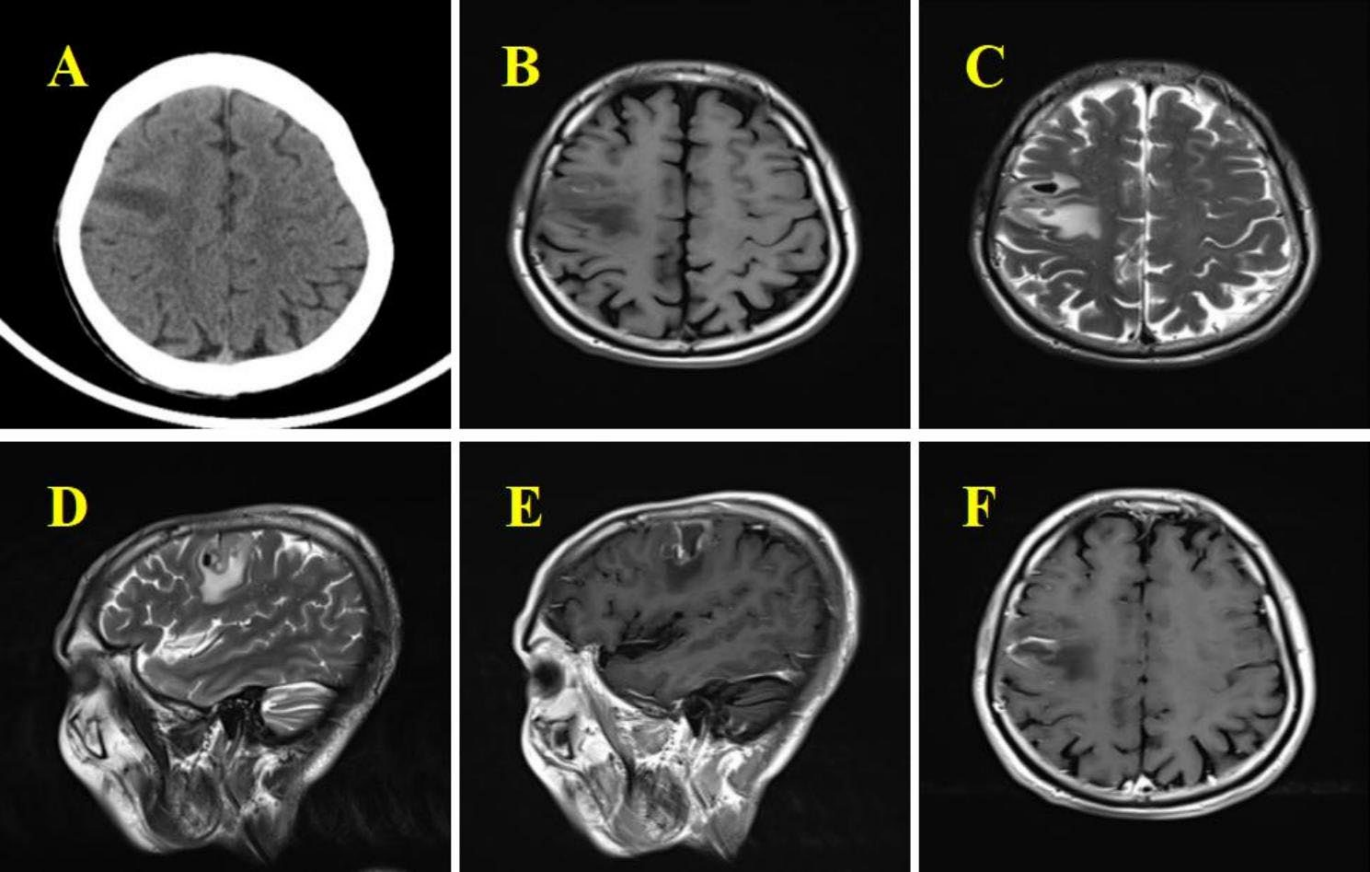
Imaging data of the patient. A. Axial CT scan of the brain showed low density lesions in the right frontal lobe. B-D. Axial T1-weighted images, T2-weighted images and sagittal T2-weighted images of head MRI were shown respectively: equal/slightly longer T1 long/short T2 abnormal signals were observed in the right frontal lobe (the anterior central gyrus was dominant), and flacy-like edema signals were observed around the lesion. E-F. Are the axial and sagittal enhanced images of brain MRI respectively: the edge of the lesion was significantly enhanced in the enhanced scan

**Fig. 2:**
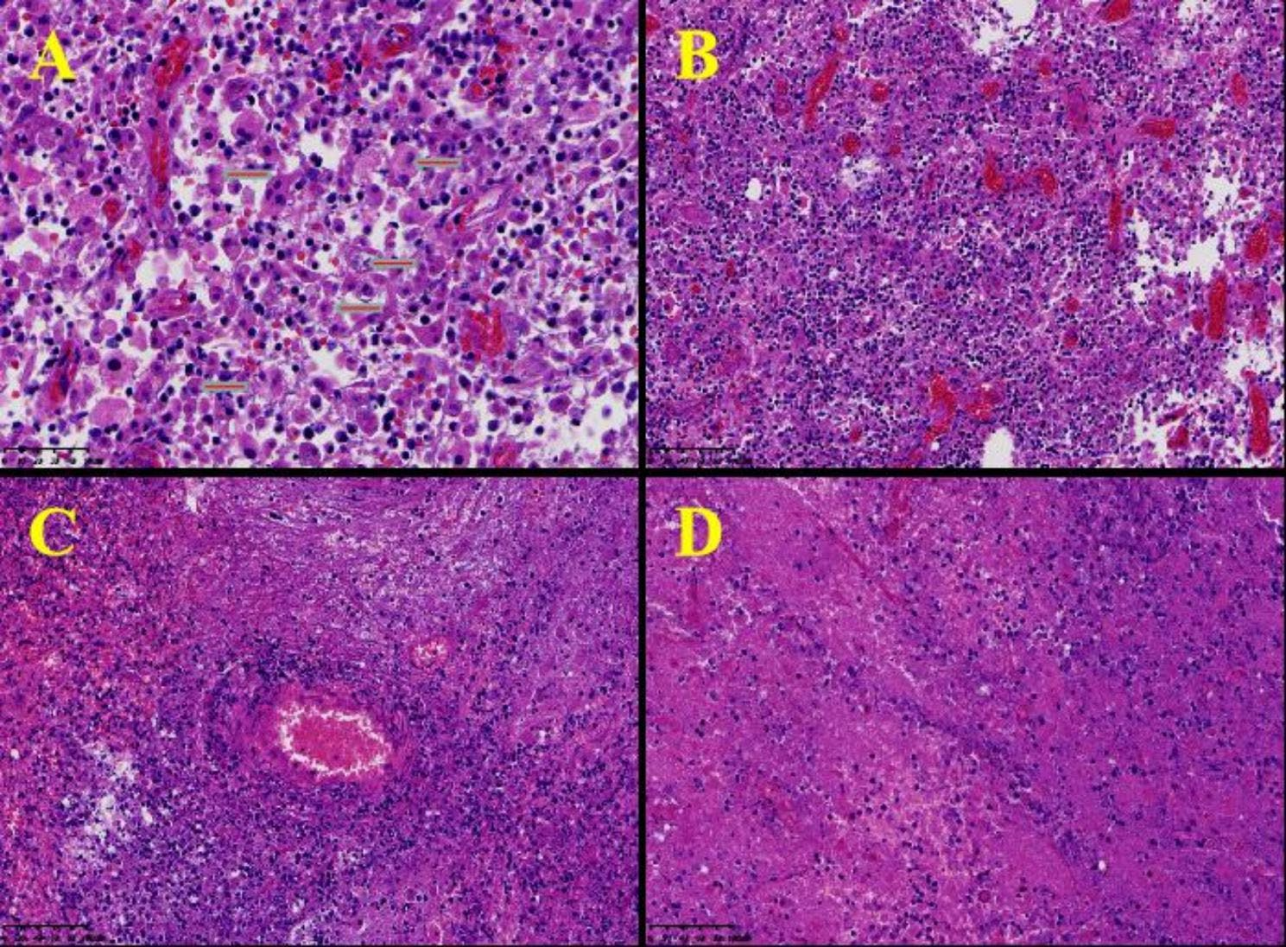
Pathological examination data of the patient. (A) A large number of diffusively distributed round trophozoites, the arrow points to the trophozoites (HE 40 × 10); (B) Inflammatory exudation dominated by lymphocytes and plasma cells (HE 20 × 10); (C) Significant cellular-like necrosis was observed in the vascular wall with infiltration of peripheral lymphocytes and plasma cells (HE 20 × 10); (D) Large sheet coagulative necrosis (HE 20 × 10)

**Fig. 3:**
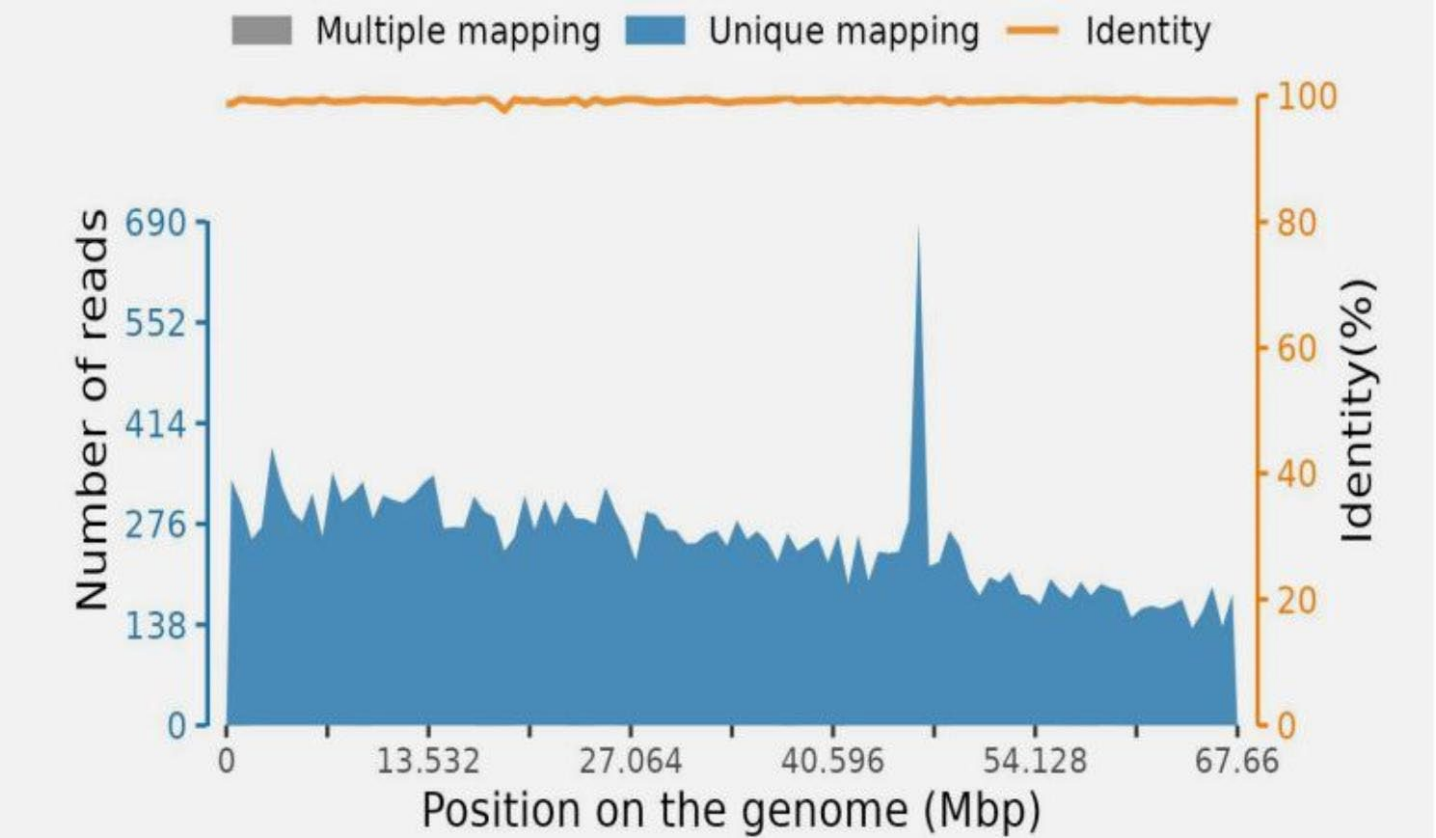
Metagenomic next-generation sequencing result of patient. The coverage chart shows the distribution of detected sequences on the genome, the blue area shows the specific sequence distribution of unique alignment, the gray area shows the sequence distribution of non-unique alignment, and the orange-red line chart shows the similarity between the detected sequence and the genome indexed sequence

In August 2021, a 61-year-old male poultry farmer from Jiangxi province of China was admitted to our hospital, He presented with moderate swelling pain in the right frontoparietal region without obvious inducement three weeks ago. He had no nausea, vomiting, limb weakness, fever, or other discomforts. Physical examination on admission showed that the temperature was 36.9℃, the pulse 86 beats/min, and the blood pressure 126/80 mmHg (1 mmHg = 0.133 kPa). The consciousness was clear, and the physical examination was cooperative. The pupils were equal and round on both sides and sensitive to light reflection. There was no other cranial nerve dysfunction, and the meningeal irritation sign was negative. There was no mass or ulcer on the whole skin. Laboratory tests showed no abnormality in blood routine and serum tumor markers screening. Head CT and MRI revealed a space-occupying lesion(2.6 cm× 1.4 cm× 0.8 cm)in the right frontal lobe(Fig. 1A-F). Based on clinical symptoms and imaging findings, the initial clinical diagnosis was a malignant tumor in the right frontal lobe with a high likelihood of high-grade astrocytoma. Resection of the right frontal mass lesion is performed under general anesthesia. Intraoperative findings: the surface of the cortex of the right frontal lobe was light yellow, hard and tough when touched. When the cortex was cut open, yellow lesions were found, which were tough in texture, fragile in part, without obvious envelope, poor blood supply, cystic changes and old bleeding in the lesions. Microscopically, it was separated along the edema zone around the focus until total removal. After the drainage tube was routinely placed, the incision was closed layer by layer. He was routinely treated for dehydration to reduce intracranial pressure after the operation. Postoperative light microscopy showed no definite malignant tumor components in the lesion tissue, locally large flake coagulated necrosis and cellular-like necrosis of the blood vessel wall, and infiltrating inflammatory cells around the blood vessels with a majority of lymphocytes and plasma cells, and a large number of round trophozoites in the background of focal bleeding (Fig. 2A-D). The pathological diagnosis of the lesion was inflammatory granulomatous lesions with extensive necrosis, suggesting amoeba infection. Subsequently, mNGS(the cost of the molecular test : USD: $1500) of cerebrospinal fluid indicated *Balamuthia mandrillaris* (sequence reads 25,386, MGISEQ-200 platform, https://www.mgitech.cn/products/instruments_info/14/). The resulting high-quality sequencing data were compared to the Microbial Genome Database (https://www.ncbi.nlm.nih.gov/data-hub/genome/?taxon=66526) to identify microorganisms and showed that the total length of the *Balamuthia mandrillaris* covering the genome was 1,229,795 (bp), the coverage was 1.8177%, and the average depth was 1.03 X(Fig. 3). The patient was diagnosed with *Balamuthia* GAE and was treated with azithromycin, fluconazole, flucytosine, and trimethoprim-sulfamethoxazole. The headache was relieved within four weeks after surgery. Unfortunately, the outcomes of the treatment weren’t successful as the condition subsequently progressed from the fifth week after surgery. Further treatment was waived, and discharge was requested and he eventually died.​.

## Discussion and conclusions

*Balamuthia mandrillaris* is a free-living amoeba found in water, air, or dust. It is pathogenic in humans and may initially enter the body through the skin or lungs, skin and systemic disseminated infections [2]. *Balamuthia mandrillaris* enters the blood circulation system, moves with the blood in multiple organs in the body. Eventually, it crosses the blood-brain barrier and migrates to the brain, causing subacute or chronic inflammatory damage to brain tissue. The resulting necrotizing granulomatous inflammation is known as *Balamuthia* GAE [2]. GAE caused by *Balamuthia mandrillaris* is rare in clinical practice and no exact incidence. Although about 300 or more cases of *Balamuthia* GAE have been reported worldwide, few clinicians know about *Balamuthia* GAE. In one study, it was found that some patients with Balamuthia GAE were immunocompromised [1]. The age of onset is not specific and has been reported in children from 4 months to 91 years old, but the case is particular in Peru, where more than half of the patients are children [3]. Its clinical symptoms are associated with inflammatory stimulation and granulomas formed from inflammatory lesions. Common clinical manifestations in reported cases are headaches and focal neurological dysfunction, other neurological symptoms such as epilepsy and psychiatric abnormalities can also present. Some infected patients often develop extrencephalic manifestations first, among which the most common is painless plaque or ulcer on the limb and face skin, and neurological symptoms occur 2–4 years later [4]. These clinical manifestations are not clearly specific to the diagnosis of *Balamuthia* GAE. Most patients have insidious onset and rapid progression and death within a few days and months of onset. Head imaging examination is helpful in the diagnosis of *Balamuthia* GAE, Head imaging examination showed [5–8] the images of patchy low-density lesions with blurred boundaries and a certain degree of space-occupying signs. MRI images show a mixture of slightly high or high signals on T1-weighted imaging, high signal on T2-weighted imaging, high signal on diffusion-weighted imaging, necrosis, and cystic degeneration in the central part, varying degrees of edema, and distinct mass effects. Contrast-enhanced MRI shows a flower ring enhancement, and some showed uneven enhancement. Due to the low incidence, non-prominent clinical symptoms, hidden epidemiological history, diversity of intracranial inflammatory lesions, low positive rate and narrow coverage of routine etiological examination, and unfamiliarity of clinicians with the disease, misdiagnosis and missed diagnosis are still likely to occur in clinical settings even after comprehensive imaging evaluation and etiological examination. The patient was an elderly male with subacute onset, no history of basic diseases such as diabetes, good nutritional status, no fever in the course of the disease, and only presented with headache symptoms. Conventional laboratory testing indicators failed to indicate the presence of pathogen infection, and the lesions on head CT and MRI images were very similar to brain tumors. This patient was diagnosed with a high-grade astrocytoma. Subsequently, the patient underwent surgery and the nature of the lesion was determined by histopathology, where trophozoites was found in the inflammatory lesion. Necrotizing granulomatous encephalitis is the basic pathological change of GAE [1.5.8]. Under light microscopy, pathological examination revealed inflammatory exudations consisting mainly of lymphocytes and plasma cells, accompanied by large coagulation necrosis and focal hemorrhage. A large number of circular trophozoites are seen in the background. The trophozoites is round and well-delimited, with slightly eosinophilic cytoplasm, visible vacuoles, and a round nucleus in the center. The presence of trophozoites in diseased tissue is an important basis for pathological diagnosis of GAE. The morphology of trophozoites is very similar to that of tissue cells, which is one of the reasons why the disease can easily be undiagnosed. Another prominent feature is the tendency of the trophozoites to erode blood vessels, with most of the vessels in the lesion showing vasculitis-like changes, with significant cellulose-like necrosis and lymphocyte and plasma cell infiltration in the vessel walls. The pathological results of the lesion were consistent with the above diagnosis of GAE in this case. Further mNGS detection of diseased tissue and cerebrospinal fluid found *Balamuthia mandrillaris*. After detecting all nucleic acid sequences of the specimen, microorganisms were compared and identified in the genomic reference database, and it was determined that the responsible microorganism was *Balamuthia mandrillaris*, the final pathological diagnosis was *Balamuthia* GAE.

Balamuthia GAE is a rare and highly fatal infection for which there is no effective treatment plan in clinical practice. Currently, treatment research comes mostly from case reports, and the number of cases is very limited. The U.S. Centers for Disease Control and Prevention recommends pentamidine, ethosulfonate, miltefosine, fluconazole, flucytosine, sulfadiazine, and clarithromycin as core agents for the treatment of *Balamuthia* GAE, but the mortality remains extremely high [9]. The literature reports that the treatment with miltefosine in individual cases has also achieved good clinical results [10]. But a continued follow-up is necessary to observe whether there is a possibility of long-term recurrence, and further in-depth research is needed to accumulate treatment experience. As to whether surgical intervention is required, we believe that surgical removal of inflammatory lesions may be considered when the patient has severe intracranial hypertension, signs of cerebral hernia, or progressive neurological dysfunction. Surgical removal may slow the progression of intracranial hypertension or, to some extent, prevent the spread of inflammatory granulomas and may also provide a pathological basis for diagnosis. In this case, the patient underwent surgical resection in combination with medication, and headache symptoms improved to some extent in the short term after the surgery, but the patient’s condition eventually worsened. Presumably, the cause may be related to delayed diagnosis, late initiation of treatment, and the lack of some drugs.

This case was misdiagnosed as high-grade astrocytoma before surgery, and our experience and lessons learned from the diagnosis and treatment of this case are: (1) Diagnostic reports from imaging should not be overly relied upon during treatment. Although imaging can help guide the direction of diagnosis, more detailed medical history and thorough clinical examination are required and combined with a patient’s past and epidemiological histories and other ancillary tests. We repeatedly asked for the patient’s relevant medical history in detail. It is difficult to pinpoint exactly what exposure led to this infection. The patient had previously suffered frequent scratches on the dorsal skin of the lower limbs from poultry without any treatment, and this was the most likely route of occult extracranial infection with *Balamuthia mandrillaris*, it may also be infected through the respiratory tract and nasal mucosa, but there is no direct etiology evidence. (2) The lack of basic knowledge and clear understanding of the epidemic spectrum of infections in this specialty suggests that we should improve our understanding of some rare pathogens. The recognition of these waterborne diseases is helpful for the diagnosis and differential diagnosis of central nervous system lesions. (3) This case fully demonstrates the great potential of mNGS detection in infectious diseases. For unexplained encephalitis, especially encephalitis caused by rare or emerging pathogens, mNGS should be properly used to help in confirming the diagnosis when conventional bacteriological and pathological techniques fail to identify the infection. Although the mNGS detection method does not provide drug susceptibility information, it should still be used as an important complement to conventional pathogen detection. (4) When a head MRI shows irregular or annular enhancement, clinicians should not easily diagnose common diseases such as brain tumors. Although *Balamuthia* GAE accounts for only a small proportion of intracranial infections, it should be considered in the differential diagnosis.

## Data Availability

The datasets used and/or analysed during the current study available from the corresponding author on reasonable request.
